# Efficacy of a pharmacist-managed diabetes clinic in high-risk diabetes patients, a randomized controlled trial - “Pharm-MD”: Impact of clinical pharmacists in diabetes care

**DOI:** 10.1186/s12902-022-00983-y

**Published:** 2022-03-16

**Authors:** Alexandra Halalau, Melda Sonmez, Ahsan Uddin, Patrick Karabon, Zachary Scherzer, Scott Keeney

**Affiliations:** 1 General Internal Medicine Division, Beaumont Health, 3601 W 13 Mile Rd, Royal Oak, MI USA; 2 Oakland University William Beaumont School of Medicine, Rochester, MI USA; 3 Internal Medicine Department, Beaumont Health, Royal Oak, MI USA; 4 Medicine- Pediatrics Department, Beaumont Health, Royal Oak, MI USA; 5 Office of Research, Oakland University William Beaumont School of Medicine, Rochester, MI USA; 6 Apogee Physicians, Erie, Philadelphia, USA

**Keywords:** Diabetes, Pharmacy, Hemoglobin A1c, Managed care

## Abstract

**Background:**

Diabetes mellitus affects 13% of American adults. To address the complex care requirements necessary to avoid diabetes-related morbidity, the American Diabetes Association recommends utilization of multidisciplinary teams. Research shows pharmacists have a positive impact on multiple clinical diabetic outcomes.

**Methods:**

Open-label randomized controlled trial with 1:1 assignment that took place in a single institution resident-run outpatient medicine clinic. Patients 18–75 years old with type 2 diabetes mellitus and most recent HbA1c ≥9% were randomized to standard of care (SOC) (continued with routine follow up with their primary provider) or to the SOC + pharmacist-managed diabetes clinic PMDC group (had an additional 6 visits with the pharmacist within 6 months from enrollment). Patients were followed for 12 months after enrollment. Data collected included HbA1c, lipid panel, statin use, blood pressure control, immunization status, and evidence of diabetic complications (retinopathy, nephropathy, neuropathy). Intention-to-treat and per-protocol analysis were performed.

**Results:**

Forty-four patients were enrolled in the SOC + PMDC group and 42 patients in the SOC group. Average decrease in HbA1c for the intervention compared to the control group at 6 months was − 2.85% vs. -1.32%, (*p* = 0.0051). Additionally, the odds of achieving a goal HbA1c of ≤8% at 6 months was 3.15 (95% CI = 1.18, 8.42, *p* = 0.0222) in the intervention versus control group. There was no statistically significant difference in the remaining secondary outcomes measured.

**Conclusions:**

Addition of pharmacist managed care for patients with type 2 diabetes mellitus is associated with significant improvements in HbA1c compared with standard of care alone. Missing data during follow up limited the power of secondary outcomes analyses.

**Trial registration:**

ClinicalTrials.gov, ID:NCT03377127; first posted on 19/12/2017.

**Supplementary Information:**

The online version contains supplementary material available at 10.1186/s12902-022-00983-y.

## Background

Diabetes mellitus affects approximately 13% of American adults and is represented by type 2 diabetes in 90–95% of cases [1]. The incidence of diabetes is projected to increase 165% by 2050, as compared with 2000 [2]. To care for this ever-growing population of patients with complex care requirements, the American Diabetes Association (ADA) recommends use of multidisciplinary teams, for which clinical pharmacists are an integral part [3].

A recent systematic review involving 36 studies on pharmacist interventions in 5671 patients with diabetes mellitus reported greater improvements in hemoglobin A1c, blood pressure, cholesterol and body mass index as compared with physician-only clinic models. Though only 3 of the studies reported cost analyses, the overall cost was found to favor pharmacist interventions [4]. A larger, more recent network meta-analysis showed pharmacist-based diabetes education and care lead to an average HbA1c reduction of 0.86% (95% CI -0.983, − 0.727%; *p* < 0.001) [5]. Despite this evidence, the optimal setting, structure, and the frequency of pharmacist visits in the care of patients with type 2 diabetes mellitus remains unknown.

Given the demonstrated benefit of the pharmacist-based approach, our hospital established a pharmacist clinic model. The uniqueness of our pharmacy-managed diabetes clinic (PMDC) was the focus of educational visits on patient identified goals and barriers to improve diabetic control. Preliminary retrospective data demonstrated a decrease of 3.2% versus 1.2% in the hemoglobin A1c at 6 months in the PMDC intervention versus a control group [6]. We aim to prospectively assess the impact of a PMDC model on diabetes core measures in patients with high-risk diabetes mellitus at 6 months and 1 year follow up.

Our hypothesis is that a pharmacist-managed diabetes clinic would have a significant positive impact on HbA1c and diabetes core measures and would result in a higher quality of care at a lower price.

## Methods

### Trial design

This study was an open-label, randomized controlled trial of a pharmacist-managed diabetes clinic in high-risk diabetes patients. Subjects were allocated 1:1 to either the standard of care (SOC) alone or to the standard of care plus the pharmacist-managed diabetes clinic (SOC + PMDC). More detailed information on study methodology can be found in the study protocol [7]. This study was approved by the Beaumont Health Institutional Review Board. The study was registered at ClinicalTrials.gov, ID: NCT03377127; first posted on 19/12/2017.

### Setting

This study was conducted at Beaumont Hospital in Royal Oak, Michigan. The site was an outpatient internal medicine resident clinic consisting of 60 internal medicine and 16 medicine-pediatrics residents who function as primary care physicians (PCPs) under the supervision of board-certified internal medicine and medicine-pediatric attending physicians. The clinic delivers care to more than 920 patients with diabetes.

### Participants

Eligibility criteria for this study included adult patients (18–75 years of age) with type 2 diabetes mellitus and the most recent hemoglobin A1c greater than or equal to 9%. Additionally, patients must have established care with an internal medicine or medicine-pediatric resident in the outpatient clinic. Patients were excluded if they were older than 75 years of age, if they had been previously enrolled in the PMDC within the previous 3 months or if they were documented in the electronic health record as being diagnosed with type 1 diabetes mellitus.

### Interventions

Patients were enrolled in our study over a 15 month period and were randomized to either the control group (SOC) or the intervention group (SOC + PMDC). The PMDC was directed by our clinic pharmacists, who scheduled face-to-face appointments. These visits had a focus on patient-identified goals and gaps in knowledge of their disease, providing educational opportunities to patients about their disease and concomitant comorbidities, conducting a diabetes-targeted medication reconciliation, and counseling patients on diet and exercise. The pharmacists, in collaboration with attending and resident physicians, could implement medication adjustments, refer patients to specialists (e.g.: ophthalmology for a dilated retinal examination), and offer vaccinations.

Both groups had an initial visit with their PCP and follow up visits at 3 and 6 months. The intervention (PMDC) group had 6 additional face-to-face visits with the PMDC during the 6 month study period [7]. These visits were scheduled more frequently in the first 3 months of the study period to ensure patient engagement. The initial PMDC visit was 60–90 min and subsequent visits were 30–45 min. Each visit was scheduled at plus or minus 8 days from the target date to allow for flexibility in scheduling the appointment. In order to improve adherence to the study protocol and minimize attrition, patients in both groups received monetary compensation in the form of a $15 gift card at each visit that was completed within the target appointment range.

### Outcomes

The primary outcome for our study was the change in hemoglobin A1c measurements between the two groups (intervention versus control) at 6 months from randomization. Secondary outcomes are detailed in the study protocol [7]. Outpatient visits reported included the PMDC visits for the intervention group.

### Sample size

Considering an allocation of 1:1 and expecting a mean difference of change in hemoglobin A1c of 1% between the two groups (intervention versus control) with a standard deviation (SD) of 1.5%, the sample size was estimated to be 36 per arm (a total of 72), with 80% power at a *p* < 0.05 significance. Adding 20% for attrition, the final sample size was estimated to be 86 patients. A hemoglobin A1c difference of 1% was considered clinically significant in long-term reduction of stroke, myocardial infarction, and microvascular complications [8].

### Randomization

Subjects were allocated to SOC + PMDC or SOC (1:1) via computer-generated block randomization with alternating sizes of 4. Sealed and opaque envelopes containing an identification number for each patient and the arm to which the subject was randomized were prepared by a biostatistician not involved in the study design or in the analyses. These envelopes were secured in a locked box only accessible by the research assistant.

### Blinding

Patients and physicians were not blinded as the pharmacist visits were documented in the electronic medical record (EMR). However, data collectors, data analysts, outcome assessors, and the biostatistician were all unaware of patient allocation. This was ensured by the research coordinator who entered each patient with non-identifying terms into a spreadsheet. Additionally, though patients were labeled in the electronic health record as being part of the study, group allocation was not disclosed. Patient assignment was not revealed until after completion of the statistical analysis.

### Statistical methods

Continuously measured and count variables were reported in terms of mean/average and standard deviation. Frequencies and percentages were displayed for categorical variables. Two Samples Independent T-Tests were used to compare continuously measured changes in HbA1c between groups. Chi-Square tests with accompanying Odds Ratios (OR) were used for binary secondary outcomes. Finally, univariate Negative Binomial regressions with Incidence Rate Ratios (IRR) were used to compare visit counts between the SOC + PMDC group and the SOC group. The per protocol analysis compared the SOC group plus non-adherent patients (less than 4 out of 6 PMDC visits from the SOC + PMDC group) with the SOC + PMDC group. *P*-Value < 0.05 indicates statistically significant findings and all analysis was completed in SAS 9.4 (SAS Institute Inc., Cary, NC, USA). A more comprehensive statistical analysis was previously published with the study protocol [7].

## Results

### Recruitment

**Fig. 1:**
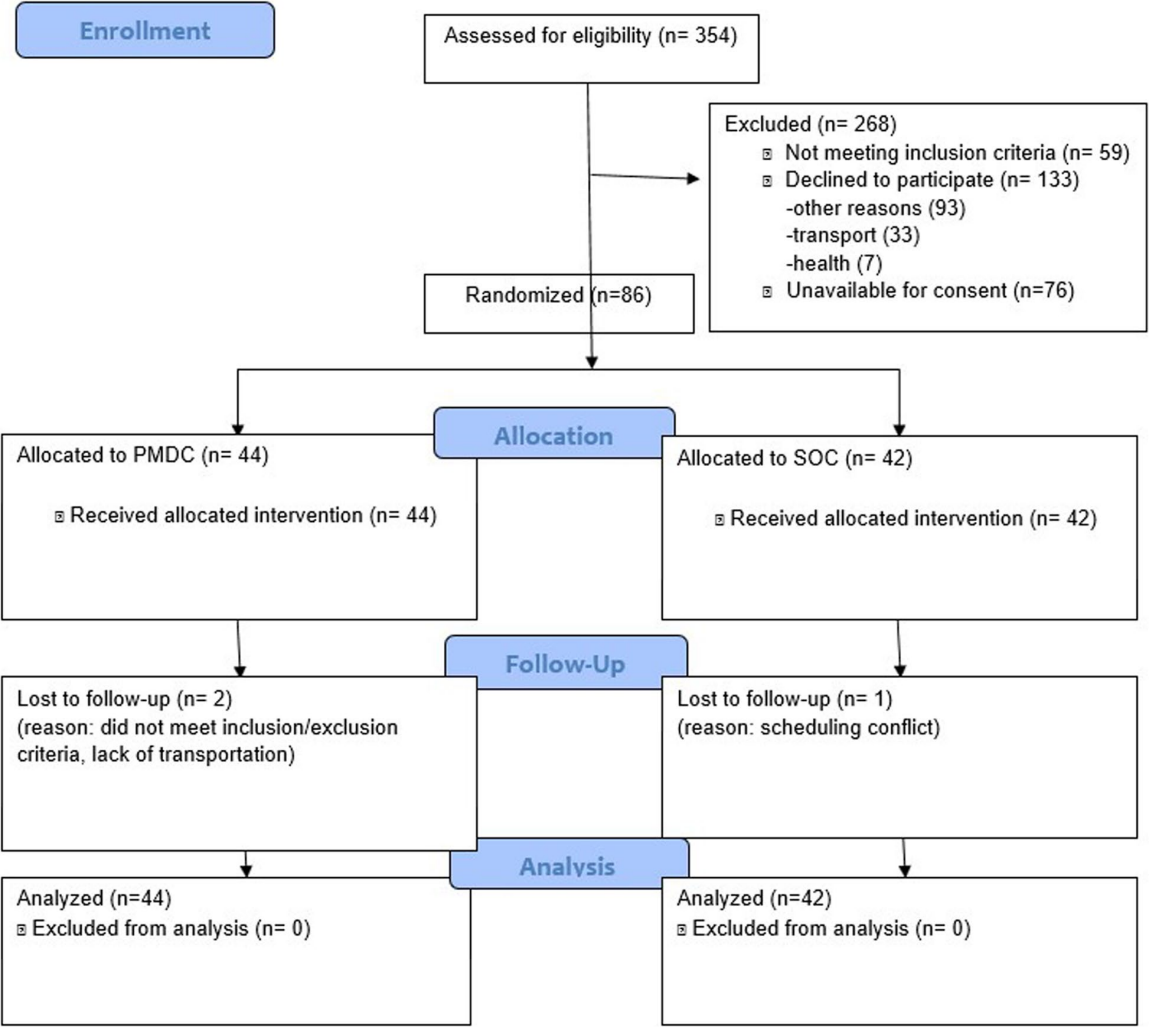
Patient enrollment flow diagram

The recruitment period took place from 02/19/2018 through 05/16/2019 with a total of 86 individuals enrolled. Patients were identified using the diabetic registry and daily clinic schedules from the EMR. Eligible patients with HbA1c greater or equal to 9% and not currently enrolled in PMDC were approached by a research assistant during the clinic visit. Reports of eligible patients scheduled for clinic appointments were reviewed once weekly. Patients meeting inclusion criteria who were overdue for PCP follow up, defined as greater than 3 months since the last visit, were contacted by phone and referred to schedule a PCP appointment. Three hundred fifty-four patients were screened, 296 patients were found eligible for the study and 245 patients were approached. Forty-four patients were enrolled in the SOC + PMDC group and 42 patients in the SOC group. Three patients dropped out (2 patients from the SOC + PMDC group and 1 patient from the SOC group). (Fig. 1).

In the PMDC+SOC group, we had 72.73% (32/44) of patients adherent to the protocol, defined as completion of at least 4 out of the 6 PMDC visits.

### Baseline data

The final study population was mostly female (58.14%) and African American (53.49%) cohort, with the remaining patient population being Caucasian (44.19%) and Hispanic (2.33%). The average age of enrolled patients was 52.69 years old and the average body mass index (BMI) was 36.66. Systolic and Diastolic Blood Pressure, on average, were 138.46 and 80.52, respectively. The average HbA1c at baseline was 11.02%, with the highest value of 18% at baseline. Among the microvascular diabetic complications, over two-thirds of patients had nephropathy (72.09%), half had neuropathy (53.49%), and just over a third had retinopathy (38.37%). Macrovascular diabetic complications were not as common at baseline (12.79%). Insulin regimens were being taken in the majority of patients (81.40%) and the remainder of the individuals exclusively used oral agents. A vast majority of enrolled patients (83.72%) also were on a statin medication for cholesterol control.

When comparing the baseline characteristics of the SOC + PMDC group and the SOC group, there were no statistically significant differences found at baseline in both the ITT analysis (all *P* > 0.05; Table 1) and PP analysis (all *P* > 0.05; Appendix Table 1).

**Table 1:** ITT Comparison of Baseline Variables between Groups

	All Patients (***n*** = 86)	SOC + PMDC (***n*** = 44)	SOC (***n*** = 42)	***P***-Value
Age				
Mean (Standard Deviation)	52.69 (8.92)	52.00 (9.67)	53.40 (8.10)	0.4684
Gender				
Female	50 (58.14%)	24 (54.55%)	26 (61.90%)	0.4892
Male	36 (41.86%)	20 (45.45%)	16 (38.10%)	
Race				
Caucasian	38 (44.19%)	17 (38.64%)	21 (50.00%)	0.5605
African American	46 (53.49%)	26 (59.09%)	20 (47.62)	
Hispanic	2 (2.33%)	1 (2.27%)	1 (2.38%)	
Body Mass Index (BMI)	**(*n*** **= 85)**	**(*n*** **= 43)**		
Mean (Standard Deviation)	36.66 (8.62)	37.54 (8.15)	35.76 (9.08)	0.3446
Systolic Blood Pressure (SBP)	**(*n*** **= 85)**		**(*n*** **= 41)**	
Mean (Standard Deviation)	138.46 (20.93)	141.55 (23.17)	135.15 (17.92)	0.1602
Diastolic Blood Pressure (DBP)	**(*n*** **= 85)**		**(*n*** **= 41)**	
Mean (Standard Deviation)	80.52 (12.30)	81.59 (13.90)	79.37 (10.37)	0.4080
Creatinine				
Mean (Standard Deviation)	1.09 (0.85)	1.07 (0.46)	1.12 (1.13)	0.7920
eGFR				
Mean (Standard Deviation)	82.71 (24.26)	82.66 (25.47)	82.76 (23.23)	0.9845
Microalbumin	**(*n*** **= 75)**	**(*n*** **= 38)**	**(*n*** **= 37)**	
Mean (Standard Deviation)	17.27 (37.45)	24.36 (46.60)	9.98 (23.31)	0.0954
Total Cholesterol	**(*n*** **= 83)**	**(*n*** **= 42)**	**(*n*** **= 41)**	
Mean (Standard Deviation)	169.54 (47.53)	168.50 (52.69)	170.61 (42.22)	0.8412
LDL	**(*n*** **= 79)**	**(*n*** **= 41)**	**(*n*** **= 38)**	
Mean (Standard Deviation)	93.94 (37.89)	93.78 (40.41)	94.11 (35.51)	0.9699
HDL	**(*n*** **= 83)**	**(*n*** **= 42)**	**(*n*** **= 41)**	
Mean (Standard Deviation)	43.67 (12.51)	42.90 (12.35)	44.46 (12.77)	0.5735
Triglycerides	**(*n*** **= 83)**	**(*n*** **= 42)**	**(*n*** **= 41)**	
Mean (Standard Deviation)	182.25 (143.49)	172.83 (126.42)	191.90 (160.12)	0.5482
Tobacco Use				
Never	42 (48.84%)	20 (45.45%)	22 (52.38%)	0.8021
Current	17 (19.77%)	9 (20.45%)	8 (19.05%)	
Former	27 (31.40%)	15 (34.09%)	12 (28.57%)	
Neuropathy				
Yes	46 (53.49%)	25 (56.82%)	21 (50.00%)	0.5263
No	40 (46.51%)	19 (43.18%)	21 (50.00%)	
Retinopathy				
Yes	33 (38.37%)	16 (36.36%)	17 (40.48%)	0.6950
No	53 (61.63%)	28 (63.64%)	25 (59.52%)	
Nephropathy				
Yes	62 (72.09%)	33 (75.00%)	29 (69.05%)	0.5384
No	24 (27.91%)	11 (25.00%)	13 (30.95%)	
Macrovascular Complications (MI, Stroke)				
Yes	11 (12.79%)	7 (15.91%)	4 (9.52%)	0.3755
No	75 (87.21%)	37 (84.09%)	38 (90.48%)	
Oral Agents Only				
Yes	28 (32.56%)	14 (31.82%)	14 (33.33%)	0.8809
No	58 (67.44%)	30 (68.18%)	28 (66.67%)	
Insulin Regimen				
Yes	70 (81.40%)	38 (86.36%)	32 (76.19%)	0.2256
No	16 (18.60%)	6 (13.64%)	10 (23.81%)	
Statin				
Yes	72 (83.72%)	35 (79.55%)	37 (88.10%)	0.2830
No	14 (16.28%)	9 (20.45%)	5 (11.90%)	

*SOC* Standard of care, *PMDC* Pharmacy managed diabetes clinic, *eGFR* Estimated glomerular filtration rate, *LDL* Low-density lipoprotein, *HDL* High-density lipoprotein, *MI* Myocardial infarction

### Primary outcome

Overall, the patients in both groups saw an average decrease in HbA1c levels of 2.12% at 6 months (*p* = < 0.0001). For the ITT analysis, the average decrease in HbA1c was significantly greater for the SOC + PMDC group as compared to the SOC group at 6 months (− 2.85% vs. -1.32%; *p* = 0.0051; Table 2*,* Appendix Fig. 1). Similarly, the PP analysis also demonstrated the decreases at 6 months (− 3.06% vs. -1.47%; *P* = 0.0111; Table 2).

**Table 2:** Change in HbA1c values at 6 and 12 months after randomization for all 4 analysis groups

	SOC + PMDC	SOC	***P***-Value
***Intent-to-Treat Analysis***			
Change in HbA1c at 6 Months	(*n* = 37) -2.85 (2.69)	(*n* = 34) -1.32 (1.66)	0.0051
Change in HbA1c at 12 Months	(*n* = 30) -2.79 (2.41)	(*n* = 31) -1.52 (1.92)	0.0259
***Intent-to-Treat with LOCF***			
Change in HbA1c at 6 Months	(*n* = 44) -2.46 (2.63)	(*n* = 42) -0.94 (1.80)	0.0024
Change in HbA1c at 12 Months	(*n* = 44) -2.42 (2.43)	(*n* = 42) -1.39 (2.08)	0.0370
***Per Protocol Analysis***			
Change in HbA1c at 6 Months	(*n* = 29) -3.06 (2.91)	(*n* = 42) -1.47 (1.66)	0.0111
Change in HbA1c at 12 Months	(*n* = 23) -2.79 (2.56)	(*n* = 38) -1.76 (1.98)	0.0816
***Per Protocol with LOCF***			
Change in HbA1c at 6 Months	(*n* = 32) -2.87 (2.84)	(*n* = 54) -1.04 (1.76)	0.0020
Change in HbA1c at 12 Months	(*n* = 32) -2.58 (2.58)	(*n* = 54) -1.53 (2.07)	0.0415

*SOC* Standard of care, *PMDC* Pharmacy managed diabetes clinic, *HgA1c* Hemoglobin A1c, *LOCF* Last observation carried forward

There was some missing data during the study. For the primary outcome, HbA1c data existed for 71 of 86 patients at 6 months (82.56% of enrolled patients) and 61 of 86 patients at 12 months (70.93%). Missing data for HbA1c data was lower in the SOC + PMDC group at 6 months (15.91% vs. 19.05%), but greater at 12 months (31.82% vs. 26.19%) as compared to the SOC group.

### Secondary outcomes

The decrease in HgA1c in between the SOC + PMDC and SOC groups was maintained at 12 months (− 2.79% vs. -1.52%; *p* = 0.0259) *(*Appendix Fig. 2). While over half (56.57%) of the patients in the SOC + PMDC group achieved the HbA1c goal of less than 8% at 6 months, only 29.41% of patients in the SOC group achieved this goal for the ITT analysis. SOC + PMDC patients had approximately 3-fold greater odds of reaching the HbA1c goal at 6 months as compared to SOC patients (OR: 3.15; *P* = 0.0222; Table 3). At 12 months for the ITT analysis, 53.33% of patients in the SOC + PMDC group had HbA1c < 8% while 35.48% of SOC patients met this goal. At 12 months, while SOC + PMDC patients had 2.08-fold greater odds of reaching the HbA1c goal, there was not enough evidence to conclude that there was a significant difference (OR: 2.08; *P* = 0.1630; Table 3). These findings were mirrored in the PP analysis (Appendix Table 3). ITT LOCF and PP LOCF analysis showed that SOC + PMDC patients had greater odds of reaching their HbA1c goal at both 6 and 12 Months (Appendix Table 2, Appendix Table 4).

**Table 3:** Secondary outcomes for Intent-to-Treat population

	**SOC + PMDC**	**SOC**	**OR (95% CI)**	***P*-Value**
HbA1c < 8.0 at 6 Months	(21 / 37) = 56.76%	(10 / 34) = 29.41%	3.15 (1.18, 8.42)	0.0222
HbA1c < 8.0 at 12 Months	(16 / 30) = 53.33%	(11 / 31) = 35.48%	2.08 (0.74, 5.81)	0.1630
Lipid Panel at 6 Months	(26 / 44) = 59.09%	(24 / 42) = 57.14%	1.08 (0.46, 2.55)	0.8548
Lipid Panel at 12 Months	(22 / 44) = 50.00%	(20 / 41) = 48.78%	1.05 (0.45, 2.46)	0.9105
Statin Therapy at 6 Months	(35 / 42) = 83.33%	(37 / 42) = 88.10%	0.68 (0.20, 2.33)	0.5346
Statin Therapy at 12 Months	(32 / 40) = 80.00%	(37 / 40) = 92.50%	0.32 (0.08, 1.33)	0.1172
Blood Pressure SBP < 140 and DBP < 90 at 6 Months	(20 / 40) = 50.00%	(27 / 39) = 69.23%	0.44 (0.18, 1.12)	0.0841
Blood Pressure SBP < 140 and DBP < 90 at 12 Months	(21 / 32) = 65.63%	(26 / 31) = 83.87%	0.37 (0.11, 1.22)	0.1027
Screening for Retinopathy at 6 Months	(35 / 44) = 79.55%	(35 / 42) = 83.33%	0.78 (0.26, 2.32)	0.6523
Screening for Retinopathy at 12 Months	(25 / 44) = 56.82%	(25 / 42) = 59.52%	0.90 (0.38, 2.11)	0.7993
Screening for Neuropathy at 6 Months	(38 / 44) = 86.36%	(39 / 42) = 92.86%	0.49 (0.11, 2.09)	0.3331
Screening for Neuropathy at 12 Months	(29 / 44) = 65.91%	(30 / 42) = 71.43%	0.77 (0.31, 1.93)	0.5818
Screening for Nephropathy at 6 Months	(34 / 44) = 77.27%	(35 / 42) = 83.33%	0.68 (0.23, 1.99)	0.4820
Screening for Nephropathy at 12 Months	(26 / 44) = 59.09%	(30 / 42) = 71.43%	0.58 (0.24, 1.42)	0.2321
Influenza vaccine at 6 Months	(24 / 44) = 54.55%	(25 / 42) = 59.52%	0.82 (0.35, 1.92)	0.6413
Influenza vaccine at 12 Months	(20 / 44) = 45.45%	(21 / 42) = 50.00%	0.83 (0.36, 1.95)	0.6732
PPSV23 vaccine at 6 Months	(27 / 44) = 61.36%	(33 / 42) = 78.57%	0.43 (0.17, 1.13)	0.0859
PPSV23 vaccine at 12 Months	(27 / 44) = 61.36%	(31 / 42) = 73.81%	0.56 (0.23, 1.41)	0.2205
**Visit Secondary Outcomes**				
	**SOC + PMDC**	**SOC**	**IRR (95% CI)**	***P*-Value**
Number of ER Visits at 6 Months	(*n* = 44) 0.68 (1.12)	(*n* = 42) 0.62 (0.99)	1.10 (0.55, 2.21)	0.7860
Number of ER Visits at 12 Months	(*n* = 44) 1.09 (1.65)	(*n* = 42) 1.05 (1.29)	1.04 (0.57, 1.89)	0.8944
Number of ER Visits for Hypo/Hyperglycemia at 6 Months	(*n* = 44) 0.00 (0.00)	(*n* = 42) 0.10 (0.37)	0.01 (0.01, 999)	0.9999
Number of ER Visits for Hypo/Hyperglycemia at 12 Months	(*n* = 44) 0.02 (0.15)	(*n* = 42) 0.12 (0.40)	0.19 (0.02, 1.84)	0.1521
Number of Inpatient Visits at 6 Months	(*n* = 44) 0.48 (0.85)	(*n* = 42) 0.38 (1.08)	1.25 (0.50, 3.16)	0.6335
Number of Inpatient Visits at 12 Months	(*n* = 44) 0.73 (1.13)	(*n* = 42) 0.71 (1.94)	1.02 (0.48, 2.18)	0.9630
Number of Outpatient Visits at 6 Months	(*n* = 44) 5.07 (2.97)	(*n* = 42) 4.55 (2.81)	1.11 (0.87, 1.43)	0.3972
Number of Outpatient Visits at 12 Months	(*n* = 44) 7.55 (4.25)	(*n* = 42) 7.60 (5.69)	0.99 (0.75, 1.31)	0.9626

*SOC* Standard of care, *PMDC* Pharmacy managed diabetes clinic, *HgA1c* Hemoglobin A1c, *SBP* Systolic blood pressure, *DBP* Diastolic blood pressure, *PPSV23* Pneumococcal polysaccharide vaccine 23, *ER* Emergency room

There was not enough evidence to conclude that there was a significant difference for any other of the secondary outcomes between the SOC + PMDC group and the SOC group in the ITT analysis (all *P* > 0.05; Table 3). In the PP analysis, the rate of Outpatient Visits at 6 Months was 38% higher in the SOC + PMDC group (IRR: 1.38; *P* = 0.0109). Otherwise, there were also no significant differences for all the other secondary outcomes (all *P* > 0.05; Appendix Tables).

For the Diabetes 39 survey, while the SOC + PMDC group had larger improvement in the overall quality of life and severity of diabetes as compared to the SOC group at 6 months, there was not enough evidence to conclude there was a significant difference between the two study arms (both *P* > 0.05; Table 4).

**Table 4:** Quality of Life Change from Baseline to 6 Months for Intent-to-Treat population

	SOC + PMDC	SOC	***P***-Value
Change in Overall Quality of Life Rating	(*n* = 23) -0.78 (2.07)	(*n* = 29) -0.31 (2.05)	0.4154
Change in Severity of Diabetes	(*n* = 23) -1.13 (2.18)	(*n* = 29) -0.28 (2.34)	0.1843

*SOC* Standard of care, *PMDC* Pharmacy managed diabetes clinic

## Discussion

### Interpretation

Our study demonstrated a significant decrease in the hemoglobin A1c (HbA1c) using a PMDC model, demonstrating that additional follow-up with clinical pharmacists for patients with diabetes mellitus is quite beneficial. Unfortunately, this study was not able to show statistically significant improvement in secondary outcomes related to other aspects of diabetic care. The clinical benefits of the combined physician-pharmacist model in care of diabetes mellitus has been previously demonstrated [3, 4]. Our study data shows a consistent benefit to the primary endpoint, HbA1c at 6-months, in the group managed by pharmacists with physicians when compared against the group managed by physicians alone. The improvement in HbA1c from baseline in the PMDC + SOC group was higher than reported in any previous studies, with a change of − 2.85 and − 2.79 at 6 months and 12 months, respectively. This data is consistent with the retrospective data analyzed in the same clinical setting [6]. All these results remained statistically significant when robust, bootstrap and jackknife methods were used to calculate standard error. These results are highly significant in the clinical settings as most classes of anti-diabetic medications have a treatment impact of approximately 0.5 to 1.25% [9]. In studies where all participants received pharmacist-based disease management, the mean improvement from baseline has varied from 0.5–2.1% at 6–12 months of follow up [10–13]. In the studies where control groups were used an improvement both in the intervention and control arm have been demonstrated during the study period [14]. In fact, the improvement in the control group seen in our study at 6 and 12 months of follow up, 1.08 and 1.81, is larger than that reported in similar studies which had approximately 1.0 [9]. This adds to the robustness of relative improvement using a pharmacist augmented care strategy.

The same data were also analyzed with respect to chances of achieving HbA1c < 8.0%, which is the goal defined by Healthcare Effectiveness Data and Information Set (HEDIS) [15]. Our results showed that the intervention group was 3.15 times more likely to achieve it at 6 months of follow up. These odds are statistically significant, however, the results were not found to be significant at 12 months follow-up. This finding is likely due to incomplete data available for analysis and reduction in number of patients for which this could be analyzed. Previous similar studies that have looked at the impact of adding a clinical pharmacist to the treatment team have also seen improvement in odds of reaching HbA1c < 8% (2.44 and 1.33 at 3- and 6-months, respectively) [9].

Other studies that looked at the implementation of pharmacist-involved collaborative care in a primary healthcare setting improved several diabetes-related outcomes over 17 months [HbA_1c_ (7.5% vs. 6.8%), low-density lipoprotein cholesterol (3.7 mmol/L vs. 2.8 mmol/L), total cholesterol (5.43 mmol/L vs. 4.34 mmol/L), and body mass index (30.42 kg/m^2^ vs. 30.17 kg/m^2^)] [16], achieved a longer time in target range for systolic blood pressure compared with control (usual care) [17] and a shorter time to therapeutic intensification and improvement in A1C goal achievement was observed with pharmacist-physician care compared with usual medical care [18]. These findings suggest that pharmacist-physician collaborative care may be one of several interventions necessary to improve the diabetes care.

Goals for diabetic care set forth by American Diabetes Association (ADA) [19] and HEDIS encompass many other factors addressing both risk management and complications related to diabetes mellitus. We included these in our analysis for patients as secondary outcomes. Of the metrics assessed, none were found to be significantly different between the groups. This also includes the number of outpatient visits per group. Despite potential for differences in compliance between the groups and a study design that would lead to more visits for the intervention group, there was no difference seen at 12 months. This unexpected finding does suggest, however, that the benefit seen from the clinical pharmacist was not simply due to the increased frequency of follow-up and instead relates to services provided at each visit.

Our study is the first to evaluate the impact of a pharmacy managed diabetes clinic focused on patient-identified goals and gaps in knowledge of their disease, and providing educational opportunities to patients about their disease and concomitant comorbidities, conducting a diabetes-targeted medication reconciliation, and counseling patients on diet and exercise. The clinical pharmacists were trained in comprehensive diabetes management, preventive therapy, and pharmacotherapy and they were running the PMDC clinic for over 6 months when our study started enrolling. Initially when the PMDC clinic started, a template in the electronic medical record was also created for consistency of the medical care provided. However, main part of the visit was left to each patient and pharmacist to address diabetes questions, concerns, medications, and preventive care that were felt to be a barrier by the patient. Our goal was to mainly provide patient driven medical care and education as each patient has different struggles and barriers when dealing with their diabetes care. We believe that this is the mechanism that could explain the improvement in diabetes care measures, as the education and care provided by the pharmacists were delivered as an answer to current struggles that the patients had, subsequently leading to improving therapeutic adherence, self-management of the disease, and motivation to change.

While we found an improvement in the PMDC + SOC group in the overall quality of life rating and in the severity of diabetes, the changes were not found to be statistically significant, mainly because of significant missing data. We have encountered unanticipated difficulty to get the patients filling out the questionnaire and mailing it back to us.

### Limitations

The limitations of our study include the small sample size, which reduced the power to detect differences in the secondary outcomes between the intervention and control group. This was further limited by the missing data not at random, despite complete follow up over the 12 month study interval. Due to the limited number of events seen in the study population, there was insufficient power to determine differences in rates of hospitalization and development of complications, which are the main drivers of cost. Therefore, we were unable to include any cost-effectiveness data in this paper. The trial was carried out in a single practice location in a suburban teaching-clinic. As a consequence, the generalizability of the data may be limited as patients with type 2 diabetes mellitus included in this study might be different from the general population. Finally, in many clinics and offices, there is likely not widespread availability of pharmacist clinics.

Additionally, though patients in the treatment arm gained durable reductions in their hemoglobin A1c values, there were extra time and monetary costs for the additional pharmacy visits. Patients in our study were reimbursed for these extra visits to incentivize follow up and avoid missing data; however, this type of reimbursement is typically absent in a “real-world” setting and thus many patients may not find these extra visits feasible. Furthermore, there are likely additional costs associated with utilization of pharmacists as well. Clinics with pharmacists would need to allocate more time for pharmacists to conduct diabetic education visits and would potentially have to hire more pharmacists to fill these roles. Additionally, patient oriented diabetic education classes may siphon time away from the other roles that pharmacists typically have.

## Conclusion

Our approach was the first to individualize the pharmacist visits based on each patient’s self-identified gaps and goals. This study adds to the current body of literature supporting the consistent addition of clinical pharmacists in the management of diabetes mellitus in the outpatient setting. Our study demonstrates robust improvements in HbA1c in the pharmacy managed diabetes clinic group and from baseline, along with greater odds of reaching HEDIS defined goal of HbA1c < 8.0% in our clinic population. The magnitude of effect is greater than that seen in similar prior studies and is maintained with prolonged follow up at 12 months. Further investigation in this area could include a comparison of different models on a broader scale to determine if one approach is superior to another. Current findings advocate for enhanced care delivery systems for this patient population.

## Supplementary Information

**Additional file 1: Appendix Figure 1.** Decrease in HbA1c Values in Intent-to-Treat Population at 6 Months HgA1c – hemoglobin A1c).

**Additional file 2: Appendix Figure 2.** Average Change in HbA1c Values in Intent-to-Treat Population (SOC – standard of care; PMDC – pharmacy managed diabetes clinic; HgA1c – hemoglobin.

**Additional file 3.**

## Data Availability

The datasets used and/or analyzed during the current study are available from the corresponding author on reasonable request.
