# Supplementary figures and images for: Efficacy of a pharmacist-managed diabetes clinic in high-risk diabetes patients, a randomized controlled trial - “Pharm-MD”: Impact of clinical pharmacists in diabetes care
Source: BMC Endocr Disord. 2022 Mar 16;22:69. doi: 10.1186/s12902-022-00983-y (PMC8925057; doi:10.1186/s12902-022-00983-y)

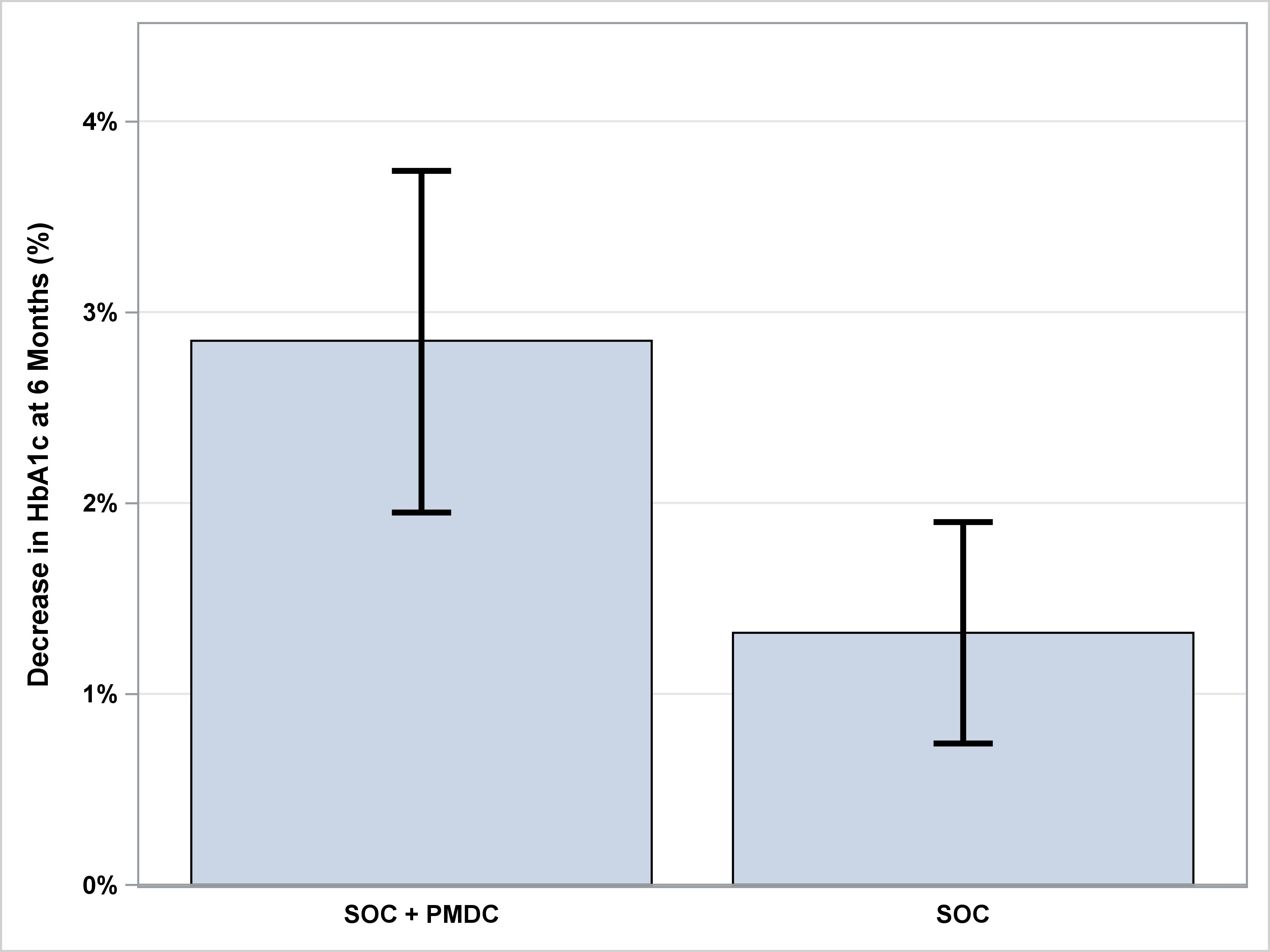

Supplement: Supplementary file 1 — Additional file 1: Appendix Figure 1. Decrease in HbA1c Values in Intent-to-Treat Population at 6 Months HgA1c – hemoglobin A1c). [file 12902_2022_983_MOESM1_ESM.jpg]

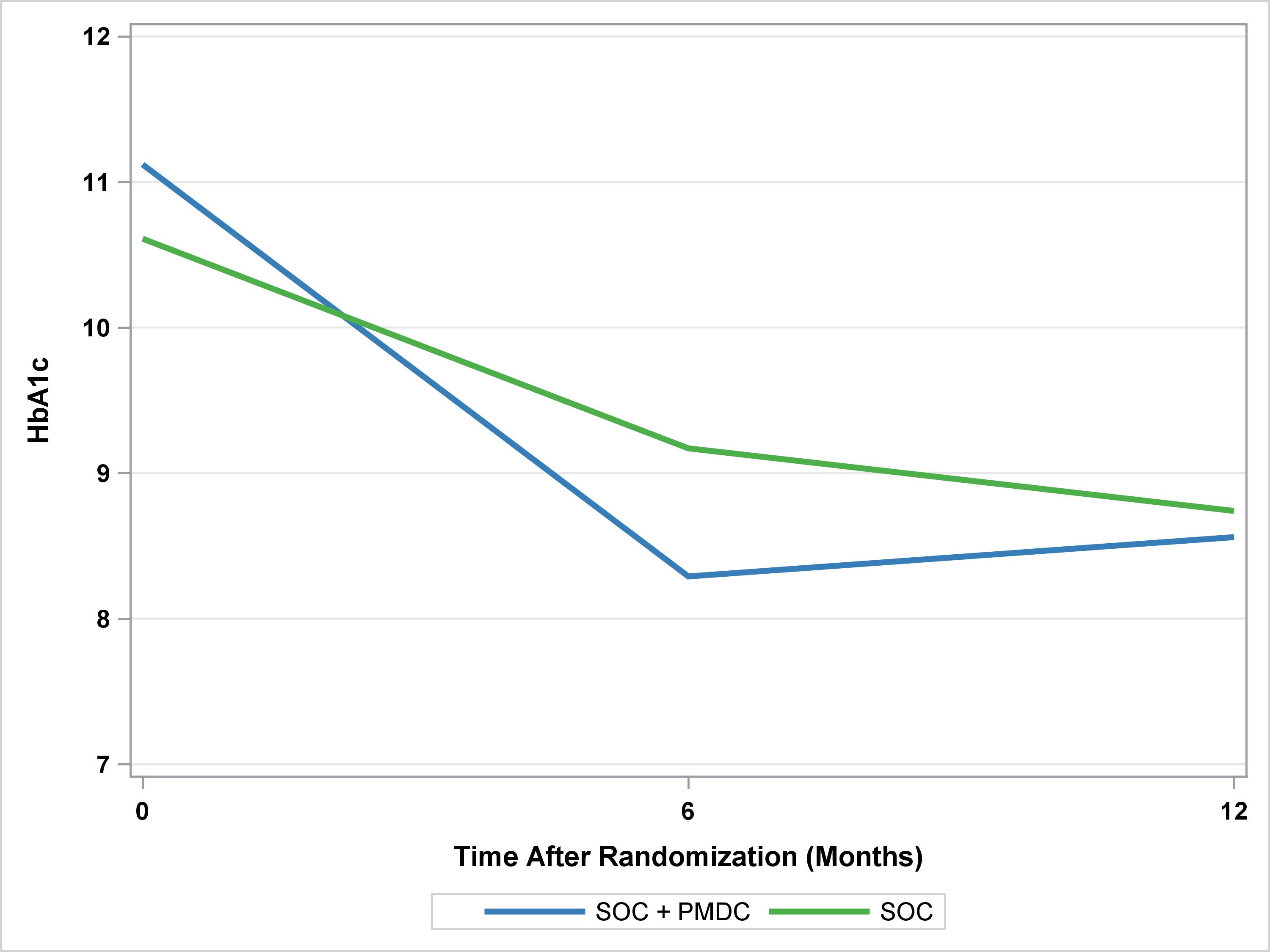

Supplement: Supplementary file 2 — Additional file 2: Appendix Figure 2. Average Change in HbA1c Values in Intent-to-Treat Population (SOC – standard of care; PMDC – pharmacy managed diabetes clinic; HgA1c – hemoglobin. [file 12902_2022_983_MOESM2_ESM.jpg]
