# Supplementary material for: Efficacy of a pharmacist-managed diabetes clinic in high-risk diabetes patients, a randomized controlled trial - “Pharm-MD”: Impact of clinical pharmacists in diabetes care
Source: BMC Endocr Disord. 2022 Mar 16;22:69. doi: 10.1186/s12902-022-00983-y (PMC8925057; doi:10.1186/s12902-022-00983-y)
Supplement: Supplementary file 3 — Additional file 3. [file 12902_2022_983_MOESM3_ESM.docx]

|  |  | **SOC + PMDC**  **(n = 32)** | **SOC**  **(n = 54)** | **P-Value** |
| --- | --- | --- | --- | --- |
| Age | |  |  |  |
|  | Mean (Standard Deviation) | 53.22 (8.45) | 52.37 (9.25) | 0.6723 |
| Gender | |  |  |  |
|  | Female | 19 (59.38%) | 31 (57.41%) | 0.8581 |
|  | Male | 13 (40.63%) | 23 (42.59%) |  |
| Race | |  |  |  |
|  | Caucasian | 13 (40.63%) | 25 (46.30%) | 0.4357 |
|  | African American | 19 (59.38%) | 27 (50.00%) |  |
|  | Hispanic | 0 (0.00%) | 2 (3.70%) |  |
| Body Mass Index (BMI) | | **(n = 31)** |  |  |
|  | Mean (Standard Deviation) | 38.24 (8.54) | 35.75 (8.61) | 0.2023 |
| Systolic Blood Pressure (SBP) | |  | **(n = 53)** |  |
|  | Mean (Standard Deviation) | 140.56 (24.42) | 137.19 (18.65) | 0.4748 |
| Diastolic Blood Pressure (DBP) | |  | **(n = 53)** |  |
|  | Mean (Standard Deviation) | 81.38 (14.08) | 80.00 (11.21) | 0.6205 |
| Creatinine | |  |  |  |
|  | Mean (Standard Deviation) | 1.09 (0.53) | 1.10 (1.00) | 0.9583 |
| eGFR | |  |  |  |
|  | Mean (Standard Deviation) | 81.19 (26.61) | 3.61 (22.96) | 0.6569 |
| Microalbumin | | **(n = 28)** | **(n = 47)** |  |
|  | Mean (Standard Deviation) | 27.44 (50.47) | 11.20 (25.70) | 0.1219 |
| Total Cholesterol | | **(n = 30)** | **(n = 53)** |  |
|  | Mean (Standard Deviation) | 168.90 (55.36) | 169.91 (43.03) | 0.9269 |
| LDL | | **(n = 29)** | **(n = 50)** |  |
|  | Mean (Standard Deviation) | 94.17 (44.09) | 93.80 (34.27) | 0.9667 |
| HDL | | **(n = 30)** | **(n = 53)** |  |
|  | Mean (Standard Deviation) | 43.07 (10.88) | 44.02 (13.43) | 0.7412 |
| Triglycerides | | **(n = 30)** | **(n = 53)** |  |
|  | Mean (Standard Deviation) | 177.67 (136.02) | 184.85 (148.76) | 0.8281 |
| Tobacco Use | |  |  |  |
|  | Never | 16 (50.00%) | 26 (48.15%) | 0.8597 |
|  | Current | 7 (21.88%) | 10 (18.52%) |  |
|  | Former | 9 (28.13%) | 18 (33.33%) |  |
| Neuropathy | |  |  |  |
|  | Yes | 21 (65.63%) | 25 (46.30%) | 0.0824 |
|  | No | 11 (34.38%) | 29 (53.70%) |  |
| Retinopathy | |  |  |  |
|  | Yes | 13 (40.63%) | 20 (37.04%) | 0.7408 |
|  | No | 19 (59.38%) | 34 (62.96%) |  |
| Nephropathy | |  |  |  |
|  | Yes | 25 (78.13%) | 37 (68.52%) | 0.3370 |
|  | No | 7 (21.88%) | 17 (31.48%) |  |
| Macrovascular Complications (MI, Stroke) | |  |  |  |
|  | Yes | 5 (15.63%) | 6 (11.11%) | 0.5446 |
|  | No | 27 (84.38%) | 48 (88.89%) |  |
| Oral Agents Only | |  |  |  |
|  | Yes | 10 (31.25%) | 18 (33.33%) | 0.8420 |
|  | No | 22 (68.75%) | 36 (66.67%) |  |
| Insulin Regimens | |  |  |  |
|  | Yes | 28 (87.50%) | 42 (77.78%) | 0.2628 |
|  | No | 4 (12.50%) | 12 (22.22%) |  |
| Statin | |  |  |  |
|  | Yes | 27 (84.38%) | 45 (83.33%) | 0.8994 |
|  | No | 5 (15.63%) | 9 (16.67%) |  |

**Appendix Table 1:** Per Protocol Baseline Characteristics (SOC – standard of care; PMDC – pharmacy managed diabetes clinic; HgA1c – hemoglobin A1c; eGFR – estimated glomerular filtration rate; LDL – low density lipoprotein; HDL – high density lipoprotein; MI – myocardial infarction)

|  | **SOC + PMDC** | **SOC** | **OR (95% CI)** | **P-Value** |
| --- | --- | --- | --- | --- |
| HbA1c < 8.0 at 6 Months | (21 / 44) = 47.73% | (10 / 42) = 23.81% | 2.92 (1.16, 7.36) | 0.0230 |
| HbA1c < 8.0 at 12 Months | (23 / 44) = 52.27% | (13 / 42) = 30.95% | 2.44 (1.01, 5.90) | 0.0472 |
| Lipid Panel at 6 Months | (26 / 44) = 59.09% | (24 / 42) = 57.14% | 1.08 (0.46, 2.55) | 0.8548 |
| Lipid Panel at 12 Months | (22 / 44) = 50.00% | (21 / 42) = 50.00% | 1.00 (0.43, 2.33) | 0.9999 |
| Statin Therapy at 6 Months | (35 / 42) = 83.33% | (37 / 42) = 88.10% | 0.68 (0.20, 2.33) | 0.5346 |
| Statin Therapy at 12 Months | (34 / 42) = 80.95% | (37 / 42) = 88.10% | 0.57 (0.17, 1.93) | 0.3692 |
| Blood Pressure SBP < 140 and DBP < 90 at 6 Months | (20 / 40) = 50.00% | (27 / 39) = 69.23% | 0.44 (0.18, 1.12) | 0.0841 |
| Blood Pressure SBP < 140 and DBP < 90 at 12 Months | (25 / 42) = 59.52% | (30 / 41) = 73.17% | 0.54 (0.21, 1.36) | 0.1910 |
| Screening for Retinopathy at 6 Months | (35 / 44) = 79.55% | (35 / 42) = 83.33% | 0.78 (0.26, 2.32) | 0.6523 |
| Screening for Retinopathy at 12 Months | (25 / 44) = 56.82% | (25 / 42) = 59.52% | 0.90 (0.38, 2.11) | 0.7993 |
| Screening for Neuropathy at 6 Months | (38 / 44) = 86.36% | (39 / 42) = 92.86% | 0.49 (0.11, 2.09) | 0.3331 |
| Screening for Neuropathy at 12 Months | (29 / 44) = 65.91% | (30 / 42) = 71.43% | 0.77 (0.31, 1.93) | 0.5818 |
| Screening for Nephropathy at 6 Months | (34 / 44) = 77.27% | (35 / 47) = 83.33% | 0.68 (0.23, 1.99) | 0.4820 |
| Screening for Nephropathy at 12 Months | (26 / 44) = 59.09% | (30 / 42) = 71.43% | 0.58 (0.24, 1.42) | 0.2321 |
| Influenza at 6 Months | (24 / 44) = 54.55% | (25 / 42) = 59.52% | 0.82 (0.35, 1.92) | 0.6413 |
| Influenza at 12 Months | (20 / 44) = 45.45% | (21 / 42) = 50.00% | 0.83 (0.36, 1.95) | 0.6732 |
| PPSV23 at 6 Months | (27 / 44) = 61.36% | (33 / 42) = 78.57% | 0.43 (0.17, 1.13) | 0.0859 |
| PPSV23 at 12 Months | (27 / 44) = 61.36% | (31 / 42) = 73.81% | 0.56 (0.23, 1.41) | 0.2205 |
|  |  |  |  |  |
| **Count Secondary Outcomes** | | | | |
|  | **SOC + PMDC** | **SOC** | **IRR (95% CI)** | **P-Value** |
| Number of ER Visits at 3 Months | (n = 44) 0.41 (0.82) | (n = 42) 0.40 (0.73) | 1.01 (0.45, 2.29) | 0.9796 |
| Number of ER Visits at 6 Months | (n = 44) 0.68 (1.12) | (n = 42) 0.62 (0.99) | 1.10 (0.55, 2.21) | 0.7860 |
| Number of ER Visits at 12 Months | (n = 44) 1.09 (1.65) | (n = 42) 1.05 (1.29) | 1.04 (0.57, 1.89) | 0.8944 |
| Number of ER Visits for Hypo/Hyperglycemia at 3 Months | (n = 44) 0.00 (0.00) | (n = 42) 0.05 (0.22) | 0.01 (0.01, 999) | 0.9932 |
| Number of ER Visits for Hypo/Hyperglycemia at 6 Months | (n = 44) 0.00 (0.00) | (n = 42) 0.10 (0.37) | 0.01 (0.01, 999) | 0.9999 |
| Number of ER Visits for Hypo/Hyperglycemia at 12 Months | (n = 44) 0.02 (0.15) | (n = 42) 0.12 (0.40) | 0.19 (0.02, 1.84) | 0.1521 |
| Number of Inpatient Visits at 3 Months | (n = 44) 0.27 (0.66) | (n = 42) 0.17 (0.44) | 1.64 (0.54, 5.00) | 0.3872 |
| Number of Inpatient Visits at 6 Months | (n = 44) 0.48 (0.85) | (n = 42) 0.38 (1.08) | 1.25 (0.50, 3.16) | 0.6335 |
| Number of Inpatient Visits at 12 Months | (n = 44) 0.73 (1.13) | (n = 42) 0.71 (1.94) | 1.02 (0.48, 2.18) | 0.9630 |
| Number of Outpatient Visits at 6 Months | (n = 44) 5.07 (2.97) | (n = 42) 4.55 (2.81) | 1.11 (0.87, 1.43) | 0.3972 |
| Number of Outpatient Visits at 12 Months | (n = 44) 7.55 (4.25) | (n = 42) 7.60 (5.69) | 0.99 (0.75, 1.31) | 0.9626 |

**Appendix Table 2:** Secondary Outcomes in the Intent-To-Treat Population with Last Observation Carried Forward (SOC – standard of care; PMDC – pharmacy managed diabetes clinic; HgA1c – hemoglobin A1c; SBP – systolic blood pressure; DBP – diastolic blood pressure; PPSV – pneumococcal polysaccharide vaccine; ER – emergency room)

|  | **SOC + PMDC** | **SOC** | **OR (95% CI)** | **P-Value** |
| --- | --- | --- | --- | --- |
| HbA1c < 8.0 at 6 Months | (17 / 29) = 58.62% | (14 / 42) = 33.33% | 2.83 (1.07, 7.54) | 0.0370 |
| HbA1c < 8.0 at 12 Months | (13 / 23) = 56.52% | (14 / 38) = 36.84% | 2.23 (0.78, 6.40) | 0.1367 |
| Lipid Panel at 6 Months | (19 / 32) = 59.38% | (31 / 54) = 57.41% | 1.08 (0.45, 2.64) | 0.8584 |
| Lipid Panel at 12 Months | (16 / 32) = 50.00% | (26 / 53) = 49.06% | 1.04 (0.43, 2.50) | 0.9328 |
| Statin Therapy at 6 Months | (28 / 32) = 87.50% | (44 / 52) = 84.62% | 1.27 (0.35, 4.63) | 0.7142 |
| Statin Therapy at 12 Months | (24 / 30) = 80.00% | (45 / 50) = 90.00% | 0.44 (0.12, 1.61) | 0.2165 |
| Blood Pressure SBP < 140 and DBP < 90 at 6 Months | (17 / 32) = 53.13% | (30 / 47) = 63.83% | 0.64 (0.26, 1.60) | 0.3425 |
| Blood Pressure SBP < 140 and DBP < 90 at 12 Months | (16 / 23) = 69.57% | (31 / 40) = 77.50% | 0.66 (0.21, 2.11) | 0.4874 |
| Screening for Retinopathy at 6 Months | (28 / 32) = 87.50% | (42 / 54) = 77.78% | 2.00 (0.59, 6.83) | 0.2688 |
| Screening for Retinopathy at 12 Months | (20 / 32) = 62.50% | (30 / 54) = 55.56% | 1.33 (0.55, 3.26) | 0.5285 |
| Screening for Neuropathy at 6 Months | (30 / 32) = 93.75% | (47 / 54) = 87.04% | 2.23 (0.44, 11.5) | 0.3358 |
| Screening for Neuropathy at 12 Months | (21 / 32) = 65.63% | (38 / 54) = 70.37% | 0.80 (0.32, 2.05) | 0.6470 |
| Screening for Nephropathy at 6 Months | (27 / 32) = 84.38% | (42 / 54) = 77.78% | 1.54 (0.49, 4.87) | 0.4600 |
| Screening for Nephropathy at 12 Months | (18 / 32) = 56.25% | (38 / 54) = 70.37% | 0.54 (0.22, 1.35) | 0.1865 |
| Influenza at 6 Months | (22 / 32) = 68.75% | (27 / 54) = 50.00% | 2.20 (0.88, 5.51) | 0.0925 |
| Influenza at 12 Months | (16 / 32) = 50.00% | (25 / 54) = 46.30% | 1.16 (0.48, 2.78) | 0.7396 |
| PPSV23 at 6 Months | (21 / 32) = 65.63% | (39 / 54) = 72.22% | 0.73 (0.29, 1.88) | 0.5203 |
| PPSV23 at 12 Months | (21 / 32) = 65.63% | (37 / 54) = 68.52% | 0.88 (0.35, 2.22) | 0.7820 |
|  |  |  |  |  |
| **Count Secondary Outcomes** | | | | |
|  | **SOC + PMDC** | **SOC** | **IRR (95% CI)** | **P-Value** |
| Number of ER Visits at 3 Months | (n = 32) 0.34 (0.65) | (n = 54) 0.44 (0.84) | 0.77 (0.33, 1.84) | 0.5601 |
| Number of ER Visits at 6 Months | (n = 32) 0.53 (0.92) | (n = 54) 0.72 (1.12) | 0.74 (0.35, 1.54) | 0.4142 |
| Number of ER Visits at 12 Months | (n = 32) 0.88 (1.13) | (n = 54) 1.19 (1.65) | 0.74 (0.39, 1.38) | 0.3435 |
| Number of ER Visits for Hypo/Hyperglycemia at 3 Months | (n = 32) 0.00 (0.00) | (n = 54) 0.04 (0.19) | 0.01 (0.01, 999) | 0.9721 |
| Number of ER Visits for Hypo/Hyperglycemia at 6 Months | (n = 32) 0.00 (0.00) | (n = 54) 0.07 (0.33) | 0.01 (0.01, 999) | 0.9999 |
| Number of ER Visits for Hypo/Hyperglycemia at 12 Months | (n = 32) 0.03 (0.18) | (n = 54) 0.09 (0.35) | 0.34 (0.03, 3.42) | 0.3582 |
| Number of Inpatient Visits at 3 Months | (n = 32) 0.22 (0.49) | (n = 54) 0.22 (0.60) | 0.98 (0.31, 3.08) | 0.9784 |
| Number of Inpatient Visits at 6 Months | (n = 32) 0.41 (0.61) | (n = 54) 0.44 (1.13) | 0.91 (0.35, 2.39) | 0.8548 |
| Number of Inpatient Visits at 12 Months | (n = 32) 0.66 (0.79) | (n = 54) 0.78 (1.89) | 0.84 (0.39, 1.83) | 0.6681 |
| Number of Outpatient Visits at 6 Months | (n = 32) 5.81 (2.87) | (n = 54) 4.22 (2.76) | 1.38 (1.08, 1.76) | 0.0109 |
| Number of Outpatient Visits at 12 Months | (n = 32) 8.25 (4.04) | (n = 54) 7.15 (5.47) | 1.15 (0.87, 1.53) | 0.3236 |

**Appendix Table 3:** Secondary Outcomes in the Per Protocol Population (SOC – standard of care; PMDC – pharmacy managed diabetes clinic; HgA1c – hemoglobin A1c; SBP – systolic blood pressure; DBP – diastolic blood pressure; PPSV – pneumococcal polysaccharide vaccine; ER – emergency room)

|  | **SOC + PMDC** | **SOC** | **OR (95% CI)** | **P-Value** |
| --- | --- | --- | --- | --- |
| HbA1c < 8.0 at 6 Months | (17 / 32) = 53.13% | (14 / 54) = 25.93% | 3.24 (1.29, 8.15) | 0.0126 |
| HbA1c < 8.0 at 12 Months | (19 / 32) = 59.38% | (17 / 54) = 31.48% | 3.18 (1.28, 7.90) | 0.0127 |
| Lipid Panel at 6 Months | (19 / 32) = 59.38% | (31 / 54) = 57.41% | 1.08 (0.45, 2.64) | 0.8584 |
| Lipid Panel at 12 Months | (16 / 32) = 50.00% | (27 / 54) = 50.00% | 1.00 (0.42, 2.40) | 0.9999 |
| Statin Therapy at 6 Months | (28 / 32) = 87.50% | (44 / 52) = 84.62% | 1.27 (0.35, 4.63) | 0.7142 |
| Statin Therapy at 12 Months | (26 / 32) = 81.25% | (45 / 52) = 86.54% | 0.67 (0.21, 2.22) | 0.5167 |
| Blood Pressure SBP < 140 and DBP < 90 at 6 Months | (17 / 32) = 53.13% | (30 / 47) = 63.83% | 0.64 (0.26, 1.60) | 0.3425 |
| Blood Pressure SBP < 140 and DBP < 90 at 12 Months | (20 / 32) = 62.50% | (35 / 51) = 68.63% | 0.76 (0.30, 1.93) | 0.5659 |
| Screening for Retinopathy at 6 Months | (28 / 32) = 87.50% | (42 / 54) = 77.78% | 2.00 (0.59, 6.83) | 0.2688 |
| Screening for Retinopathy at 12 Months | (20 / 32) = 62.50% | (30 / 54) = 55.56% | 1.33 (0.55, 3.26) | 0.5285 |
| Screening for Neuropathy at 6 Months | (30 / 32) = 93.75% | (47 / 54) = 87.04% | 2.23 (0.44, 11.5) | 0.3358 |
| Screening for Neuropathy at 12 Months | (21 / 32) = 65.63% | (38 / 54) = 70.37% | 0.80 (0.32, 2.05) | 0.6470 |
| Screening for Nephropathy at 6 Months | (27 / 32) = 84.38% | (42 / 54) = 77.78% | 1.54 (0.49, 4.87) | 0.4600 |
| Screening for Nephropathy at 12 Months | (18 / 32) = 56.25% | (38 / 54) = 70.37% | 0.54 (0.22, 1.35) | 0.1865 |
| Influenza at 6 Months | (22 / 32) = 68.75% | (27 / 54) = 50.00% | 2.20 (0.88, 5.51) | 0.0925 |
| Influenza at 12 Months | (16 / 32) = 50.00% | (25 / 54) = 46.30% | 1.16 (0.48, 2.78) | 0.7396 |
| PPSV23 at 6 Months | (21 / 32) = 65.63% | (39 / 54) = 72.22% | 0.73 (0.29, 1.88) | 0.5203 |
| PPSV23 at 12 Months | (21 / 32) = 65.63% | (37 / 54) = 68.52% | 0.88 (0.35, 2.22) | 0.7820 |
|  |  |  |  |  |
| **Count Secondary Outcomes** | | | | |
|  | **SOC + PMDC** | **SOC** | **IRR (95% CI)** | **P-Value** |
| Number of ER Visits at 3 Months | (n = 32) 0.34 (0.65) | (n = 54) 0.44 (0.84) | 0.77 (0.33, 1.84) | 0.5601 |
| Number of ER Visits at 6 Months | (n = 32) 0.53 (0.92) | (n = 54) 0.72 (1.12) | 0.74 (0.35, 1.54) | 0.4142 |
| Number of ER Visits at 12 Months | (n = 32) 0.88 (1.13) | (n = 54) 1.19 (1.65) | 0.74 (0.39, 1.38) | 0.3435 |
| Number of ER Visits for Hypo/Hyperglycemia at 3 Months | (n = 32) 0.00 (0.00) | (n = 54) 0.04 (0.19) | 0.01 (0.01, 999) | 0.9721 |
| Number of ER Visits for Hypo/Hyperglycemia at 6 Months | (n = 32) 0.00 (0.00) | (n = 54) 0.07 (0.33) | 0.01 (0.01, 999) | 0.9999 |
| Number of ER Visits for Hypo/Hyperglycemia at 12 Months | (n = 32) 0.03 (0.18) | (n = 54) 0.09 (0.35) | 0.34 (0.03, 3.42) | 0.3582 |
| Number of Inpatient Visits at 3 Months | (n = 32) 0.22 (0.49) | (n = 54) 0.22 (0.60) | 0.98 (0.31, 3.08) | 0.9784 |
| Number of Inpatient Visits at 6 Months | (n = 32) 0.41 (0.61) | (n = 54) 0.44 (1.13) | 0.91 (0.35, 2.39) | 0.8548 |
| Number of Inpatient Visits at 12 Months | (n = 32) 0.66 (0.79) | (n = 54) 0.78 (1.89) | 0.84 (0.39, 1.83) | 0.6681 |
| Number of Outpatient Visits at 6 Months | (n = 32) 5.81 (2.87) | (n = 54) 4.22 (2.76) | 1.38 (1.08, 1.76) | 0.0109 |
| Number of Outpatient Visits at 12 Months | (n = 32) 8.25 (4.04) | (n = 54) 7.15 (5.47) | 1.15 (0.87, 1.53) | 0.3236 |

**Appendix Table 4:** Secondary Outcomes in the Per Protocol Population with Last Observation Carried Forward (SOC – standard of care; PMDC – pharmacy managed diabetes clinic; HgA1c – hemoglobin A1c; SBP – systolic blood pressure; DBP – diastolic blood pressure; PPSV – pneumococcal polysaccharide vaccine; ER – emergency room)
